# Diphylleia Grayi-Inspired Intelligent Temperature-Responsive Transparent Nanofiber Membranes

**DOI:** 10.1007/s40820-023-01279-z

**Published:** 2024-01-04

**Authors:** Cengceng Zhao, Gaohui Liu, Yanyan Lin, Xueqin Li, Na Meng, Xianfeng Wang, Shaoju Fu, Jianyong Yu, Bin Ding

**Affiliations:** Shanghai Frontier Science Research Center of Advanced Textiles, College of Textiles, Donghua University, Shanghai, 201620 People’s Republic of China

**Keywords:** Biomimetic, Transparent, Nanofibrous membrane, Temperature response, Phase change materials

## Abstract

Nanofiber membranes (NFMs) have become attractive candidates for next-generation flexible transparent materials due to their exceptional flexibility and breathability. However, improving the transmittance of NFMs is a great challenge due to the enormous reflection and incredibly poor transmission generated by the nanofiber-air interface. In this research, we report a general strategy for the preparation of flexible temperature-responsive transparent (TRT) membranes, which achieves a rapid transformation of NFMs from opaque to highly transparent under a narrow temperature window. In this process, the phase change material eicosane is coated on the surface of the polyurethane nanofibers by electrospray technology. When the temperature rises to 37 °C, eicosane rapidly completes the phase transition and establishes the light transmission path between the nanofibers, preventing light loss from reflection at the nanofiber-air interface. The resulting TRT membrane exhibits high transmittance (> 90%), and fast response (5 s). This study achieves the first TRT transition of NFMs, offering a general strategy for building highly transparent nanofiber materials, shaping the future of next-generation intelligent temperature monitoring, anti-counterfeiting measures, and other high-performance devices.

**Supplementary Information:**

The online version contains supplementary material available at 10.1007/s40820-023-01279-z.

## Highlights

- Temperature-responsive transparent nanofiber membranes were successfully fabricated using a straightforward and widely applicable method.
- The temperature-responsive nanofiber membranes exhibit a lower reaction temperature (~ 37 °C) and higher transmittance (> 90%).
- The prepared temperature-responsive transparent nanofiber membranes exhibited a short response temperature time (~ 5 s), and remarkable stability

**Supplementary Information:**

The online version contains supplementary material available at 10.1007/s40820-023-01279-z.

## Graphical abstract

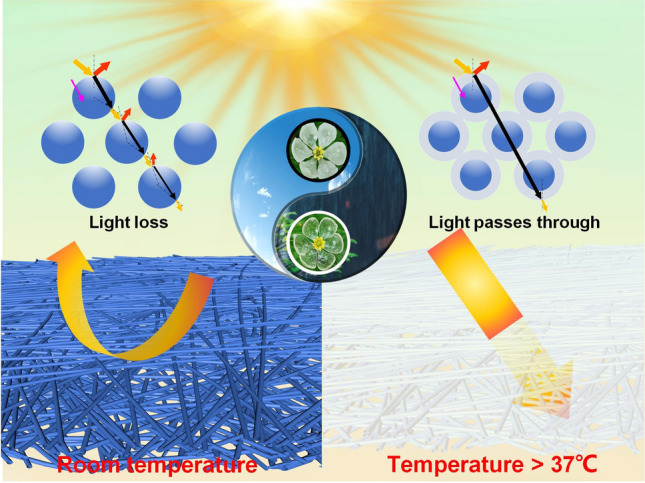

## Introduction

The demand for optical transparent materials is rapidly increasing across various industries, with applications ranging from transparent windows [1–4], flexible displays [5–8], wound dressings [9, 10], and air filtration [11, 12]. However, traditional glass and ceramics [13], known for their optical clarity and hardness, are rigid and lack the necessary mechanical toughness for practical use cases that require flexible deformation. Consequently, there is an increasing demand for the development of flexible, transparent materials that can be readily tailored to fit a range of applications. The development of such materials is essential for modern industries that require materials that are both mechanically robust and environmentally resistant, in addition to being optically transparent. There are numerous applications for flexible transparent materials, ranging from biomedical [14, 15] and environmental engineering [16–18] to the development of flexible sensors [19, 20] and electrochemical [21–23] components. However, their development has been hindered by weak mechanical properties, low breathability, and high costs. Therefore, developing transparent materials with flexible and breathable properties is still a great challenge to date.

Electrospinning nanofiber membranes (NFMs) are becoming a candidate with an appeal for transparent materials due to their unique properties. However, the porosity [24], thickness [25], fiber diameter [26], and interfacial refractive index [27] (n) of the NFMs cause unfavorable light refraction and reflection, causing extremely low light transmittance (Fig. S1). To tackle this issue, significant research efforts have been dedicated to enhancing the effective transmission of light while reducing refraction and scattering via nanofibers [28]. The development of optimized electrospinning techniques, novel materials, and advanced surface treatments is currently underway to produce NFMs with improved light transmission properties. These efforts may lead to the development of flexible and breathable transparent NFMs that could have applications across various industries [29–31]. As an example of recent advancements in the field, Li et al. [32] achieved a high level of transmittance and excellent mechanical properties by combining nonsolvent-induced phase separation and liquid injection when preparing cellulose acetate-based membranes. Similarly, Yang et al. [33] created polymer composite sheets with high transmittance by curing liquid silica nanoparticle-filled precursor, which could then be cut, folded, pyrolyzed, and sintered to produce three-dimensional glass. These techniques showcase the potential for the creation of advanced composite materials with high transmittance and mechanical properties, which could have vast applications across various industries. However, these reported transparent fibrous membranes exhibit low porosity and complex fabrication processes. For example, Wang et al. [24] examined the porosity-transmittance relationship in transparent nanofiber membranes. They observed transmittance values of 0.1%, 78%, 83%, and 89% at porosities of 98.1%, 27.7%, 23.2%, and 10.2%, respectively. This demonstrated a decrease in porosity with increasing transmittance. Thus, to further fulfill the practical demand, the design, and construction of transparent fibrous membranes with simple preparation, high light flux, superior flexibility, and excellent mechanical properties are urgently needed.

Herein, we presented the first-ever smart temperature-responsive transparent nanofiber membranes (TRT-NFMs), designed through the process of electrospinning and electrospray [34–36]. Inspired by Diphylleia grayi, we simulated the cell structure by using the composite structure of polyurethane (PU, n = 1.51) and eicosane (C_20_H_42_, n = 1.4425). With several advantages, including simplicity and controllability, the utilization of electrospinning and electrospray techniques presents a straightforward approach to the preparation of TRT-NFMs. As a result, a TRT nanofiber membrane with high mechanical strength and temperature sensitivity was obtained. This innovative approach provides a proof of concept for the advancement of transparent NFMs with smart properties, which are suitable for use across a diverse array of applications.

## Experimental Section

### Materials

PU (W_n_ = 14,000) was bought from BASF Polyurethane Specialties Co., Ltd. Eicosane was obtained from Macklin. N, N-Dimethylformamide (DMF), N, N, N-trimethyl-1-dodecanaminium bromide (DTAB), and acetone were supplied by Shanghai Aladdin Chemistry Co., Ltd.

### Fabrication of NFMs

#### Preparation of Electrospinning NFMs

To obtain precursor solution by dissolving PU in DMF solvents with optimized additives. Then, stirring was conducted for 8 h to achieve a homogenized solution for obtaining the PU electrospinning solution. Following that, The HZ-12 electrospinning machine (Qingdao Nuokang Enviro. Prot. Tech. Co., Ltd. China) was utilized. To control the uniformity of the membranes, the sliding table was moved circularly within 20 cm at a velocity of 100 mm min^−1^ and applied a continuous DC voltage of 25 kV. The spinning distance between the syringe needle and the receiving roller was 25 cm. The preparation process of nanofibers was carried out in the laboratory with a relative humidity of ~ 50 ± 10% and a temperature of ~ 25 ± 5 °C.

#### Preparation of Electrospray NFMs

A constant weight ratio of 1:200, 1:100, and 1:50 was maintained between eicosane and acetone. After stirring in a water bath with a magnetic stirrer at 40 °C for 30 min, a homogeneous and transparent electrospray solution was obtained. These solutions were then designated as PU-C_20_-1, PU-C_20_-2, and PU-C_20_-3. The detailed solution compositions are summarized in Table S1. Using the electrospun nanofiber membrane prepared according to section 2.2.1 as the substrate, in order to ensure the smooth progress of the electrospray process, the electrospray experiment was conducted at a certain ambient temperature. The preparation instrument and parameters are the same as in 2.2.1.

### Materials Characterization and Measurement

#### Characterization

The surface morphology of the relevant fibrous membranes was investigated using a field emission scanning electron microscope (FE-SEM, SU5000, Japan). The thickness of the samples was tested by thickness equipment (CHY-C2, Lab-think Instruments Co., Ltd. China). A capillary flow porometer (CFP-1100AX, Porous Materials Inc. USA) was used to evaluate the porous structures. The porosities were computed using the following formula:

1 $$Porosity = \frac{{V_{d} - V_{p} }}{{V_{d} }} \times 100\%$$

where $V_{d}$ and $V_{p}$ represent the volumes of the polymer and the electrospun membranes, respectively. The air permeability was measured by the YG461E automatic air permeability tester. The mechanical properties were tested using dynamic mechanical analysis (Suzhou ShengTe Tech. Co., Ltd. China). Thermal gravimetric analysis (TGA) and derivative thermogravimetric curve (DTG) were conducted under a nitrogen atmosphere using a TGA2-8860 analyzer. DSC analysis was performed on a DSC250 instrument (TA Instruments, USA). Experiments were performed under the N_2_ atmosphere. To erase thermal history, the samples were heated from 25 to 60 °C with a heating rate of 10 °C min^−1^ and then maintained at 60 °C for 5 min. Following that, the samples were cooled at a rate of 10 °C min^−1^ to − 60 °C and maintained there for 5 min before being scanned from − 60 to 60 °C at a rate of 10 °C min^−1^. The reheating curves were used to generate the DSC curves. The chemical structure of NFMs was assessed using Fourier-transform infrared (FTIR) spectroscopy with a Nicolet-8700 spectrometer (Thermo Fisher Scientific Inc., USA). X-ray diffractometry (XRD, D8 Advanced Bruker, Germany) was employed to determine the crystallinity. The XRD patterns were analyzed with diffraction angles ranging from 10° to 90° at a scan rate of 5° min^−1^.

#### Polarizing Optical Microscope (POM) Measurement

The XPL-3230 POM (Shanghai Teelen Optical Instruments Co., Ltd. China) equipped with a hot stage was used to monitor the crystalline size of the eicosane phase in PU-C_20_-3 during the cyclic heating–cooling process.

#### Optical Performance Measurement

The samples were heated from 30 to 50 °C at a rate of 6 °C min^−1^ using a hot stage. A 3-min hold at the maximum temperature was performed, followed by cooling at a rate of 6 °C min^−1^ controlled by a developed temperature control system. The dynamic transmittance and absorbance of the samples were evaluated using a BIM-6001 spectrometer (manufactured by Bro-light Photoelectric Tech. Co., Ltd. China) with an XD-301 halogen light source (manufactured by WENCE Photoelectric Tech. Co., Ltd. China). Static transmittance test using UV–vis Photometer (Summit Instrument Manufacturing Co., Ltd. China). Haze measurements were conducted using a TH110 haze meter (Cai Pu Tech. Co., Ltd. China).

## Results and Discussion

### Design and Construction of TRT-NFMs

**Fig. 1:**
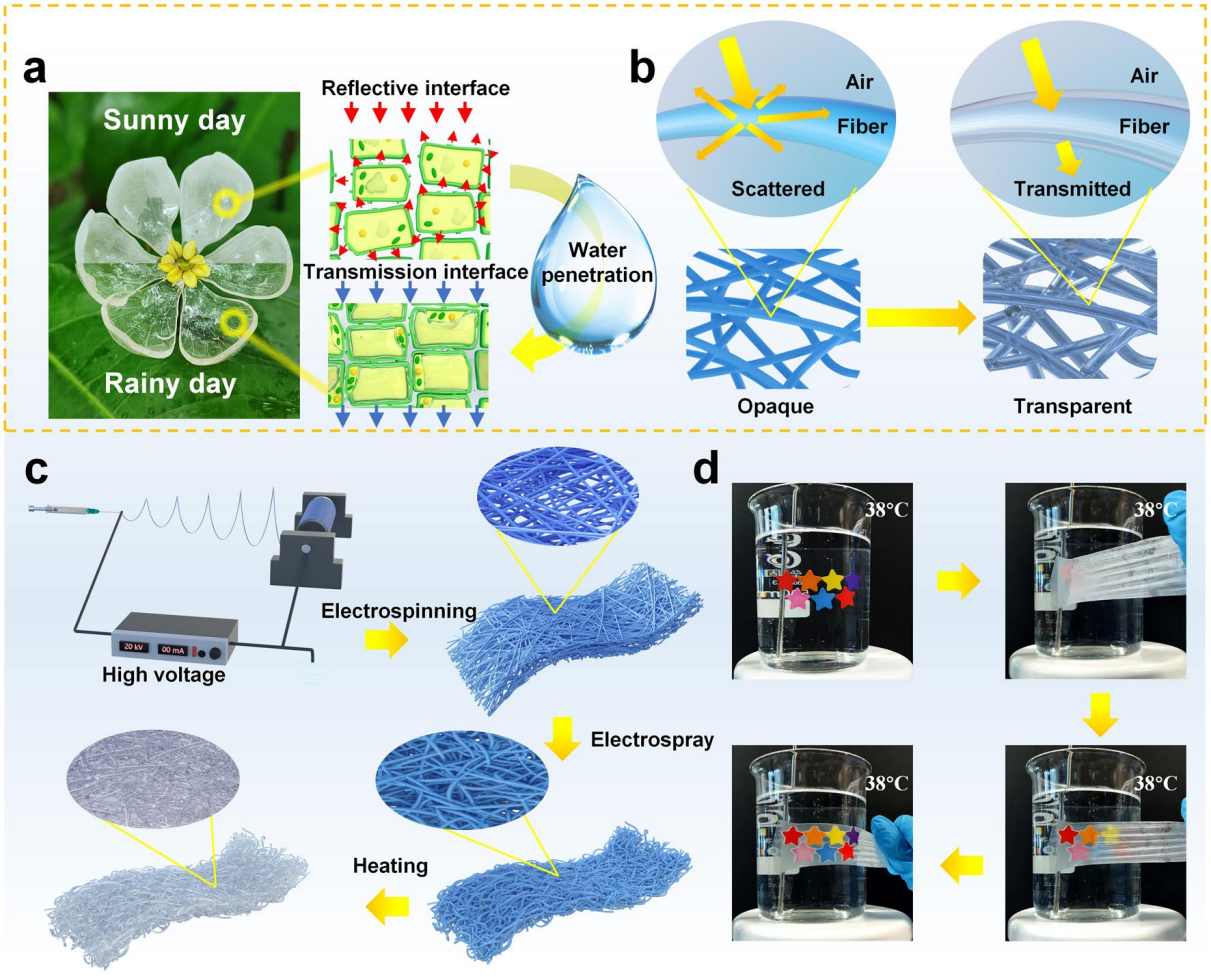
**a** The different states of Diphylleia grayi on sunny and rainy days. Diphylleia grayi shown is the model flower. **b** Mechanism of transmittance in NFMs based on bionics. **c** Schematic illustrating the fabrication of TRT-NFMs. **d** Photograph images of the temperature response of transparent NFMs working on the surface of a 38° beaker

The Diphylleia grayi, which naturally boasts a white hue, showcases a rather extraordinary ability: it can transform from an opaque state to a highly transparent one [37]. This unique quality, it turns out, is because the rain causes water to flood the gaps between the plant's cells (as shown in Fig. 1a). Drawing inspiration from this natural wonder, we introduced a temperature-responsive phase change material, eicosane [38], to fiber-based porous membranes to manipulate the direction of light propagation in the membranes (as demonstrated in Fig. 1b). In crafting our TRT-NFMs, we deliberately selected PU polymer due to its exceptional qualities, including weather resistance [39] and high elasticity [40], as well as tear resistance [41]. Unfortunately, pure PU NFMs are often incredibly opaque, which led us to consider the incorporation of eicosane to overcome this limitation. During the eicosane phase transition, which occurs around 37 °C, it transforms from a solid to a liquid state and evenly coats the PU nanofibers. The homogeneous coating of the surfaces enables the two materials to produce a surface with almost the same refractive index. This characteristic enables lossless refraction of natural light that interacts with the surface. Furthermore, the liquid eicosane's amorphous state exhibits weak refractivity and low reflection of light, which minimizes the scattering of light at the interface between air and PU nanofibers. This characteristic allows the PU TRT nanofibers to rapidly shift from opaque to transparent during the eicosane phase transition. The result of this innovative combination of materials is an incredibly versatile and adaptable membrane with a wide range of potential uses.

The process of developing the TRT-NFMs is a multi-step procedure that is visualized in detail in Fig. 1c. Firstly, an opaque PU nanofiber membrane was created through the use of the common electrospinning method. This membrane provided the foundation for the subsequent stages of the process. Next, eicosane was used to develop a nanofiber membrane with temperature-responsive transparency by uniformly coating it onto the PU nanofibers. The cutting-edge electrospray technique was utilized to accomplish this task [42]. When heated to 37 °C, the liquid eicosane underwent a rapid phase transition, forming a thick directional transmission path between the nanofibers. This process imparted transparency onto the otherwise opaque NFMs, making them highly transparent with transmittance conversion ratios of up to 25:1 (Table S2). The effectiveness of the TRT nanofiber membrane was demonstrated by placing it on the surface of a beaker filled with water heated to 38 °C. Figure 1d notably showcased the exceptional transparency responses and chromogenic chroma of the TRT fibrous membrane, captured from movie S1 in the supplementary material.

### Analysis of Morphology and Mechanical Properties

**Fig. 2:**
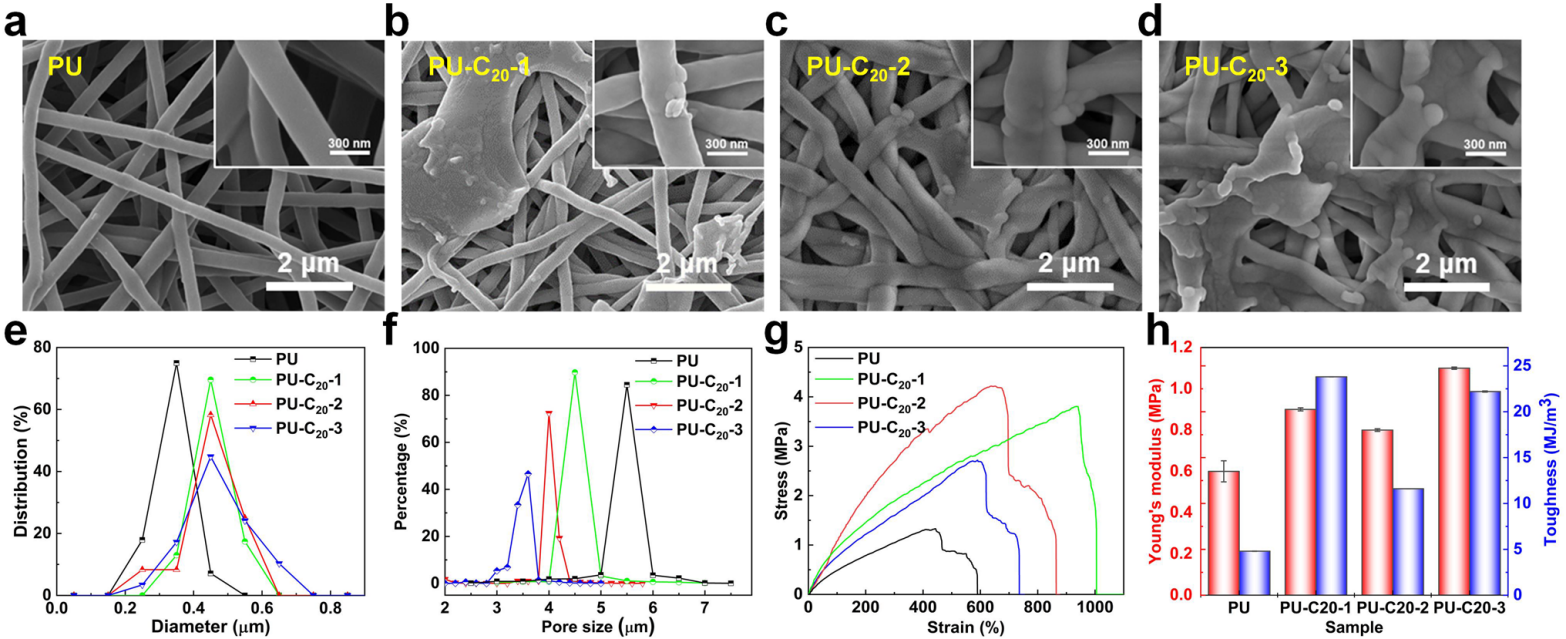
**a** SEM image of pure PU NFMs. **b**–**d** SEM images of PU NFMs with different ratios of eicosane. **e** Diameter distribution of the NFMs. **f** Pore size distribution. **g** The tensile strength of transmittance changes membranes. **h** Young's modulus and toughness

Based on the experimental phenomena in Fig. S2, the substrate fiber membrane thickness (~ 11 ± 1 μm) was determined for subsequent experiments. Figure 2a–d showcases a series of SEM images of TRT-NFMs, each with varying amounts of eicosane. Notably, when compared to pure PU NFMs, the addition of eicosane greatly improved the adhesion between the nanofibers. As anticipated, the sample with the highest eicosane content, labeled as PU-C_20_-3, retained the original porousness of the NFMs before and after the phase transition (Fig. S3). It is evident that the use of eicosane in the preparation of these NFMs results in a durable product with desirable optical properties. By carefully controlling the amount of eicosane present, researchers can achieve the ideal combination of adhesion and porosity for their specific application. In Fig. 2e, the diameter distribution information of the NFMs is presented, revealing distinctive patterns across the various samples. Specifically, the average diameter of pure PU nanofibers was evaluated at 340 nm, accounting for 75% of the sample. As the concentration of eicosane increased, an increase in the average diameter and dispersion of nanofibers was also observed. These changes were attributed to the coating of eicosane on the nanofiber surface. Significantly, the sample with the highest quantity of eicosane (PU-C_20_-3) demonstrated the greatest increase in the average nanofiber diameter, measured at 470 nm. This observation further validates the successful coating of eicosane onto the PU nanofiber surface, corroborating the SEM. This knowledge can be applied to finely adjust the composition of the nanofiber membrane, ensuring optimal performance and precise levels of transmittance.

Figures 2f and S4, S5 depict the impact of eicosane concentration on the pore size, porosity, and air permeability of the NFMs, revealing a substantial decrease in pore size, porosity, and air permeability with increasing eicosane content. Compared to the average pore size of 5.5 μm in pure PU NFMs, the average pore size of the NFMs after electrospray decreased by 1–1.9 μm. The TRT-NFMs are not only effective in maintaining the structural integrity of the porous NFMs but also exhibit remarkable mechanical properties. Specifically, the pure PU NFMs demonstrated a tensile stress of 1.33 MPa and a strain of 591%, as illustrated in Fig. 2g. With the addition of eicosane, however, the tensile stress and strain of the NFMs significantly increased. The stress increased by more than 200%, while the strain increased by more than 100%. These results confirm the outstanding mechanical properties of the TRT-NFMs when compared with pure PU NFMs. Furthermore, the pure PU NFMs demonstrated Young's modulus of 0.6 MPa and toughness of 4.8 MJ m^−3^, respectively. After electrospray with eicosane, significant improvements in Young's modulus and toughness of the NFMs were observed, as portrayed in Fig. 2h. The improvement in mechanical strength and durability of eicosane-enhanced TRT-NFMs can be attributed to the presence of a long alkyl chain in twenty leading to a toughening effect on epoxy-containing PU [43, 44], in addition, the physical crosslinking of eicosane between PU nanofibers has a significant impact on the synergistic improvement of stress, strain, and modulus [45]. The combination of toughening and cross-linking effects can significantly improve the mechanical strength and durability of TRT-NFMs. These properties make the film an excellent choice for applications that require strength, flexibility, and optical transparency.

### Thermal and Melting-Crystallization Behaviors

**Fig. 3:**
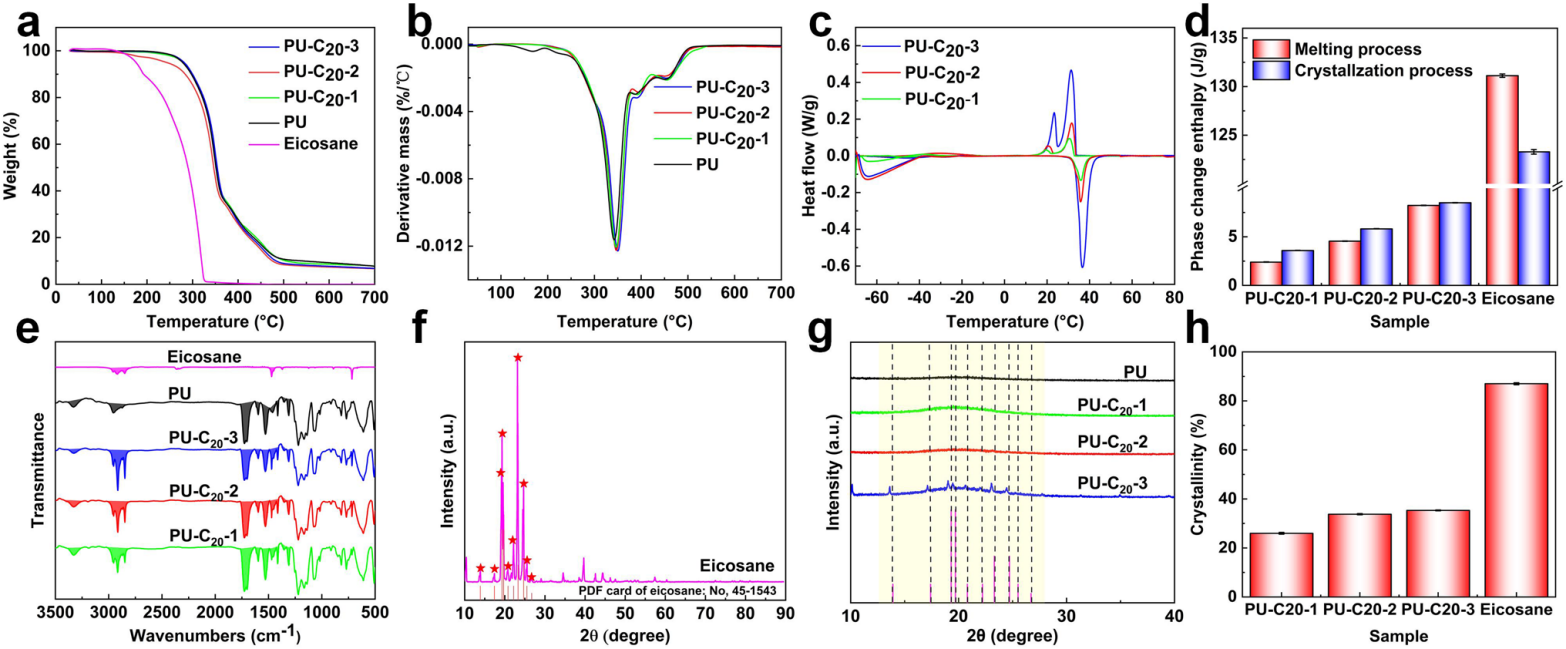
**a-b** TG and the corresponding DTG curve. **c** DSC curves and **d** corresponding histogram. **e** FTIR. **f** XRD diagram of eicosane. **g** XRD of composite NFMs and corresponding **h** Crystallinity. *A. u.* Arbitrary units, *2θ* Diffraction angle

The thermogravimetric analysis of thermosensitive transparent NFMs is a crucial area of study, particularly given its ability to shed light on the behavior of temperature-responsive polymers. As demonstrated in Figs. 3a and S6, eicosane showcases one weight loss process between 170 and 320 °C, which is due to eicosane degradation. Meanwhile, pure PU begins to lose weight at about 200 °C and becomes entirely weightless at 500 °C. It is also noteworthy that eicosane displays two crystallization peaks [46] with temperatures ranging from 21 to 33 °C and one melting peak at approximately 38 °C. These peaks could be linked to the varying crystallization temperatures of the distinct phases of eicosane (as seen in Fig. S7). After the incorporation of eicosane, the decomposition temperature and rate of the composite sample remained relatively unchanged. This observation is mainly due to the low content of eicosane in the mixture. Figures 3c and S8 showcase the differential scanning calorimetry curves of samples with differing eicosane content, as well as their corresponding enthalpy of phase transformation. Interestingly, there is a new melt crystallization peak in the presence of eicosane, indicating that its addition significantly affects the thermal characteristics of TRT-NFMs. However, the presence of PU in the mixture may influence eicosane's ability to crystallize, as seen in the steadily declining enthalpy of melting and crystallization as the quantity of eicosane decreases (Fig. 3d). These findings reveal the intricate nature of thermosensitive transparent NFMs and the importance of studying the behavior of temperature-responsive polymers, as their properties can greatly impact the physical characteristics of materials.

As shown in Fig. 3e, the characteristic peaks of the infrared spectra of PU-C_20_-1, PU-C_20_-2, and PU-C_20_-3 show specific patterns of PU: 3321 cm^−1^ for the –NH stretching mode, 2955 cm^−1^ for the –CH_2_ and –CH_3_ stretching mode, 1719 cm^−1^ for the –C=O stretching mode of amido-carbonyl, 1599 cm^−1^ for the –C_6_H_6_ skeleton vibration, 1528 cm^−1^ for the –CONH_2_ II band, and 1311 cm^−1^ for the –CONH_2_ III band. Moreover, the characteristic peaks of the –CH_2_ and –CH_3_ stretching modes of eicosane were observed at 2915 and 2848 cm^−1^, and the –CH_2_ deformation modes of eicosane were observed at 1471 and 1377 cm^−1^, respectively. Furthermore, the peak height was different with different amounts of eicosane added, which further confirmed the successful introduction of the eicosane group into the PU nanofiber membrane. By analyzing the FTIR spectra of PU-C_20_-1, PU-C_20_-2, and PU-C_20_-3, it is evident that both eicosane and PU characteristic peaks appear and have different peak intensities compared to those in the spectra of pure eicosane. This suggests that eicosane and PU have a robust interface interaction.

According to Fig. 3f, eicosane exhibits five distinctive diffraction peaks at 19.56°, 19.91°, 23.25°, 24.84°, and 25.55°, which correspond to its β crystals (010), (011), (105), (− 101), and (110), respectively. Additionally, the diffraction peaks at 10.58°, 13.91°, 39.81°, and 44.56° correspond to (003), (004), (0–22), and (207) of eicosane α crystals, respectively, which are the same as the standard PDF card numbers. Upon analyzing the XRD patterns of the NFMs in Fig. 3g, it was found that PU-C_20_-1, PU-C_20_-2, and PU-C_20_-3 contained diffraction peaks of eicosane. This suggests that eicosane was incorporated into the PU, and the characteristic peaks of eicosane were not affected. Figure 3h shows the calculation of the crystallinity, which indicates that the presence of various amounts of eicosane resulted in a gradual decrease in crystallinity compared to pure eicosane. It is likely that this decrease may affect the transmittance of the NFMs.

**Fig. 4:**
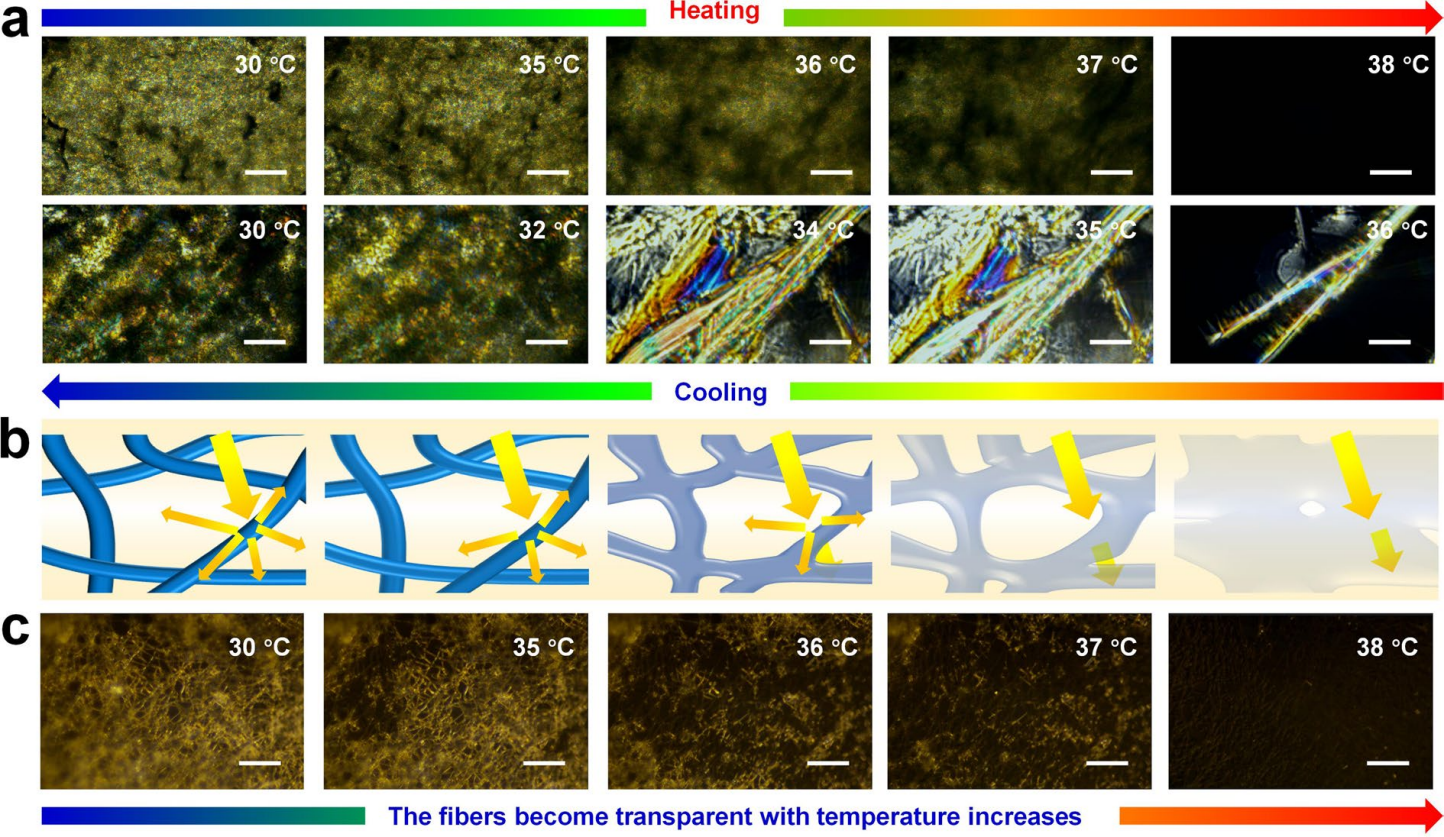
**a** POM images of eicosane upon a melting-crystallization. **b** Transparent mechanism of the NFMs during the heating process and **c** The corresponding POM photographs of the NFMs. Scale bar: 40 μm

As polymer optics and crystallization behavior are closely intertwined, it becomes imperative to delve deeper into understanding the melt**-**crystallization behavior of TRT-NFMs [47, 48]. In order to gain a comprehensive understanding of this phenomenon, polarizing optical microscopic images of eicosane throughout a melt crystallization cycle have been presented in Fig. 4a. When heated from 30 to 35 °C, eicosane crystals do not alter, but when heated further to 37 °C, the crystals melt quickly. Moreover, the cooling process does not result in crystallization until the temperature reaches 37 °C. Eicosane rapidly crystallizes when the temperature is lowered to 36 °C, and the local rapid crystallization results in intergranular cracking under tensile stress [49]. The crystalline cracks expand as the temperature drops, swiftly filling the area until they crash with one another [50]. Overall, the quick disappearance of eicosane crystals at 37 °C and their sudden appearance at 36 °C indicate high sensitivity to temperature for eicosane crystallization behavior. Studies have revealed that the grains in semi-crystalline polymers may prevent light from passing through, making the substance opaque or less transparent [51, 52]. In contrast, amorphous polymers provide lossless light transmission and thus display excellent transmittance [53, 54]. Figure 4b, c depict the microscopic images of the TRT-NFMs with eicosane added, captured from Movie S2. As the temperature increases, crystallization change caused by the change of the eicosane phase leads to a large amount of light transmission, thus achieving high transmittance.

### Optical Performance

**Fig. 5:**
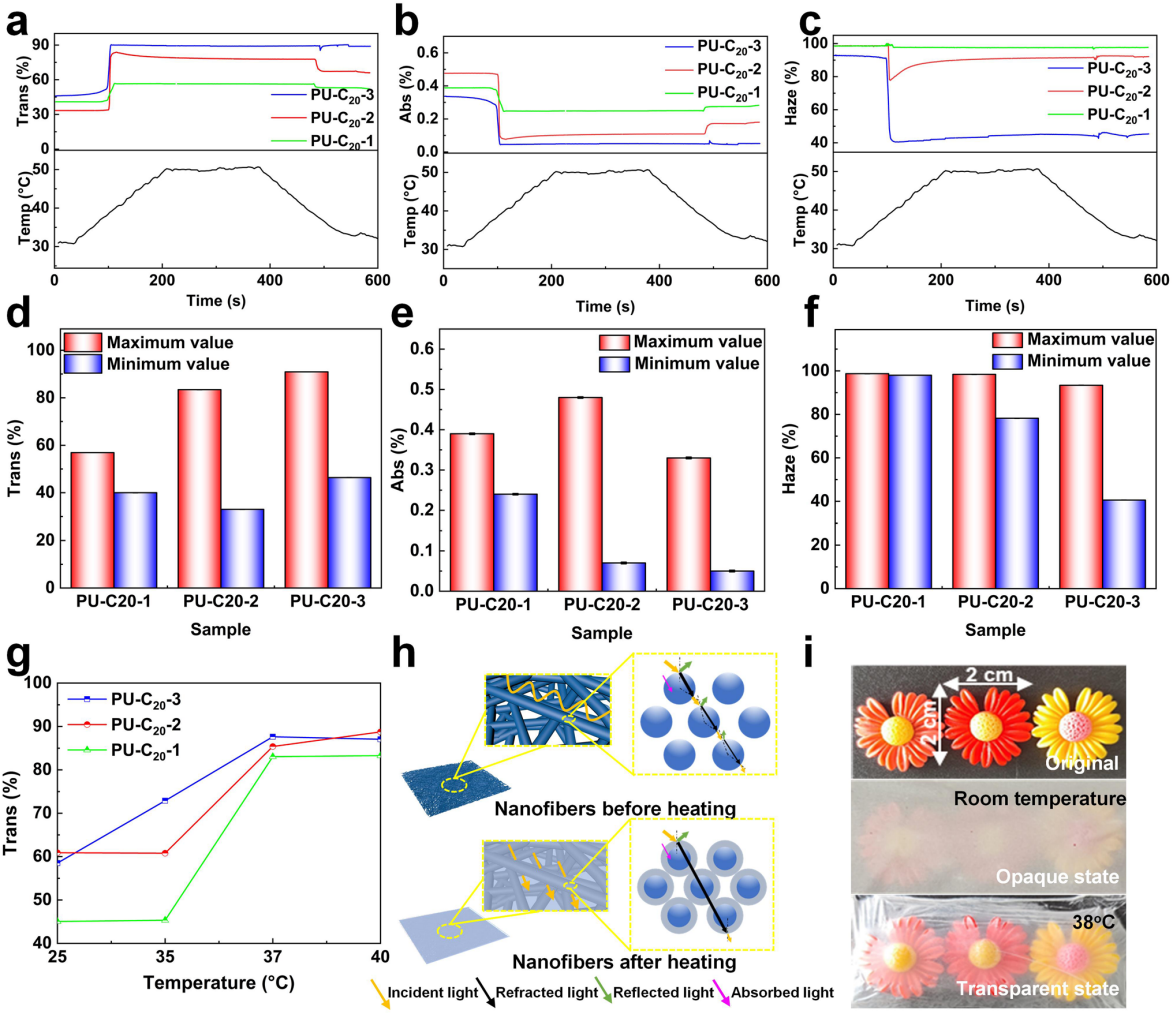
The sample's **a** transmittance, **b** absorbance and **c** haze. **d–f** The statistical extreme values obtained from a to c. **g** The transmittance of samples at 550 nm wavelength. **h** Propagation mechanism of light in the NFMs. **i** Optical photographs of NFMs in different states

Figure 5a–c presents the dynamic behavior of the temperature-responsive NFMs, PU-C_20_-1, PU-C_20_-2, and PU-C_20_-3, in terms of light transmittance, absorbance, and haze while undergoing the heating and cooling processes from 30 to 50 °C. Notably, all the samples exhibited the lowest transmittance, highest haze, and highest absorbance at 30 °C. As the temperature increased from 30 to 50 °C, light transmission increased and the conversion ratio of transmittance reached 25:1 (Tables S2, S3), while the haze and absorbance decreased across all samples. Notably, the peak values of transmittance and the lowest values of haze and absorbance occurred at approximately 38 °C and showed data stability for all three samples (Fig. S9). The detailed values of the extreme transmittance, absorbance, and haze of the temperature-responsive NFMs are presented in Fig. 5d–f. Interestingly, PU-C_20_-3 demonstrated the highest transmittance (~ 90.9%), the lowest absorbance (0.05%), and the lowest haze (~ 40.6%). However, the maximum transmittance reduced from 90.9 to 56.9%, while the maximum absorbance and haze increased from 0.33% and 93.4% to 0.48% and 98.7%, respectively, as the quantity of phase change material content added decreased. Taking PU-C_20_-3 as an example, at lower temperatures (~ 30 °C), this sample showed the maximum absorbance and haze, as well as the lowest transmittance, as expected. However, as the temperature increased, the transmittance started to increase at a swift pace at about 37 °C, while the absorbance and haze decreased rapidly. This effect was associated with the rapid phase change of eicosane. These observations offer important insights into the potential of temperature-responsive polymers to enhance the optical properties of NFMs. In addition to the optical properties, rapid response is a crucial factor in temperature-responsive polymers [55]. As depicted in Fig. 5a–c, all three samples displayed swift responsiveness within 5 s to achieve the desired transmittance transformation. Moreover, the samples exhibited high haze, which was primarily due to the concave and convex porous structure formed by the interlacing of fibers on the NFMs' surface [56]. The concave and convex porous surface causes incident light to be deflected, thus resulting in an increase in haze. We looked further into the transmittance at 550 nm visible light wavelength because it is widely known that this wavelength is the most perceptible to the human eye [57]. The outcome of our investigation, as illustrated in Fig. 5g, indicated that all three samples demonstrated a remarkable transmittance of 80% at 550 nm optical wavelength (at 37 °C). Throughout the remainder of our research, we focused primarily on the PU-C_20_-3 sample, unless stated otherwise. It is well understood that two prominent features of TRT-NFMs are chrominance and quick transmittance transition capabilities.

As illustrated in Fig. 5h, a significant quantity of light cannot penetrate the NFMs caused of the reflection and refraction of light at the interface between nanofibers and the surrounding environment. Additionally, the high porosity of all NFMs creates a significant number of air-fiber interfaces [58, 59]. Since the air and nanofiber at the interface have extremely different refractive indices, the light will be strongly reflected or refracted there, resulting in the opacity of the NFMs. However, after electrospray eicosane, the NFMs demonstrated good light transmission after heating, indicating that the amount of light reflection had been significantly reduced. This phenomenon is because the refractive index of eicosane in the liquid state is similar to that of PU nanofiber, which results in a much lower light reflection/refractive index at the interface. Figure 5i shows the temperature-responsive appearance of the transparent nanofiber membrane in different states, which is a valid proof of our theory. The TRT-NFMs prepared in this work have excellent mechanical properties and TRT conversion, opening the door for their applications in next-generation frontal temperature stickers, anti-counterfeiting stickers, vegetable greenhouses, and dimming windows.

## Conclusions

In summary, flexible NFMs that exhibit fast and effective transparent transformation capabilities were successfully developed by the researchers using electrospinning and electrospray techniques, whereby phase change materials were utilized to achieve this state transformation. The creation of NFMs with exceptional mechanical properties and uniform diameters was enabled by the electrospinning approach, serving as a valuable guide to designing transparent NFMs with desired mechanical properties. Moreover, different additions of phase change materials were experimented with using electrospray, each resulting in varying degrees of transmittance upon the temperature elevation (> 37 °C). This marks a significant improvement from the existing electrospinning technology that could only create white NFMs, making the smart transparent transformation feature of the electrospinning NFMs a novel discovery. In addition, the TRT-NFMs prepared exhibit rapid response to temperature changes and possess the advantage of a short response interval, ensuring their stability and functionality. A comprehensive approach to enhancing the light transmission of electrospinning membranes for practical applications, including anti-counterfeiting and dimming windows, among others, is offered by this research. Hence, the foundation for exploring advanced responsive and functional materials is provided by this research.

## Supplementary Information

Below is the link to the electronic supplementary material.

Supplementary file1 (PDF 746 kb)

Supplementary file2 (MOV 10712 kb)

Supplementary file3 (MOV 24615 kb)
