# Supplementary material for: Diphylleia Grayi-Inspired Intelligent Temperature-Responsive Transparent Nanofiber Membranes
Source: Nanomicro Lett. 2024 Jan 4;16:65. doi: 10.1007/s40820-023-01279-z (PMC10766919; doi:10.1007/s40820-023-01279-z)
Supplement: Supplementary file 1 — Supplementary file1 (PDF 746 kb) [file 40820_2023_1279_MOESM1_ESM.pdf]

Supporting Information for

# Diphylleia Grayi-Inspired Intelligent Temperature-Responsive Transparent Nanofiber Membranes

Cengceng Zhao[1], Gaohui Liu[1], Yanyan Lin[1], Xueqin Li[1], Na Meng[1], Xianfeng Wang[1,*], Shaoju Fu[1,*], Jianyong Yu[1], and Bin Ding[1,*]

[1] Shanghai Frontier Science Research Center of Advanced Textiles, College of Textiles, Donghua University, Shanghai 201620, P. R. China

[*]Corresponding authors. E-mail: wxf@dhu.edu.cn (Xianfeng Wang); sjfu@dhu.edu.cn (Shaoju Fu); binding@dhu.edu.cn (Bin Ding)

## S1 Supplementary Methods

### S1.1 Optical Thickness

The behavior of materials under light can be explained by optical thickness (OT), which is the ratio of the physical thickness of a material to the average distance that light travels before changing direction [S1-S4].

### S1.2 Ballistic Propagation and Multiple Scattering

Light can propagate through the material in two ways: ballistic propagation, where the majority of light propagates in the same direction as the incident light, and multiple scattering, where the light propagates in various directions. When light is in ballistic propagation and the material has a relatively low OT, the light propagates almost undisturbed, and the material appears transparent. On the other hand, in multiple scattering, when the material has a relatively high OT (> 8), the material appears opaque and white [S5-S6].

## S2 Supplementary Tables

**Table S1** Detailed compositions and concentrations of precursor solutions for the fabrication of various membranes

| Solution | 1 | 2 |
|---|---|---|
| Solvents | DMF | acetone |
| Main polymer | PU | Eicosane |
| Additive | DBAT | / |
| Main polymer concentration | 18wt% | 2wt%/1wt%/0.5wt% |
| Additive concentration | 1.5% | / |

**Table S2** The transmittance conversion ratio of samples PU-$C_{20}$-1, PU-$C_{20}$-2, PU-$C_{20}$-3

| Sample | $T_t$ | $T_0$ | Ratio |
|---|---|---|---|
| PU-$C_{20}$-1 | 55.62% | | 15:1 |
| PU-$C_{20}$-2 | 78.90% | 3.62% | 22:1 |
| PU-$C_{20}$-3 | 90.15% | | 25:1 |

**Table S3** The weight loss of different samples after heating 5 times

| Sample | Weight loss %/$m^2$ |
|---|---|
| PU | 0 |
| PU-$C_{20}$-1 | 0 |
| PU-$C_{20}$-2 | 0 |
| PU-$C_{20}$-3 | 0.17 |

## S3 Supplementary Figures

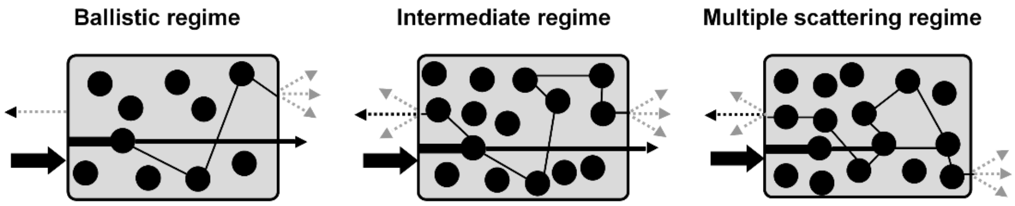


**Fig. S1** Illustration of different light-propagation regimes in disordered media: from ballistic to multiple scattering. For simplicity, the transition between different regimes is depicted by varying the filling fraction of the system [S7, S8]. Ballistic beams, specular reflection, and scattered light are represented by solid black, dotted black, and dotted gray lines, respectively. Line thickness qualitatively represents the difference in intensity between ballistic and scattered light in every regime

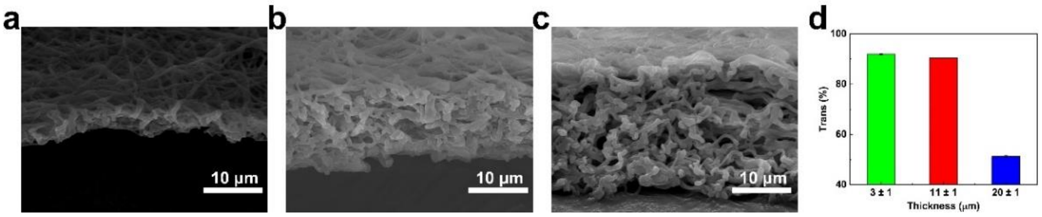


**Fig. S2** Cross-sectional SEM images of different thicknesses and transmittance. **a** 3 ± 1 μm. **b** 11 ± 1 μm. **c** 20 ± 1 μm. **d** Transmittance

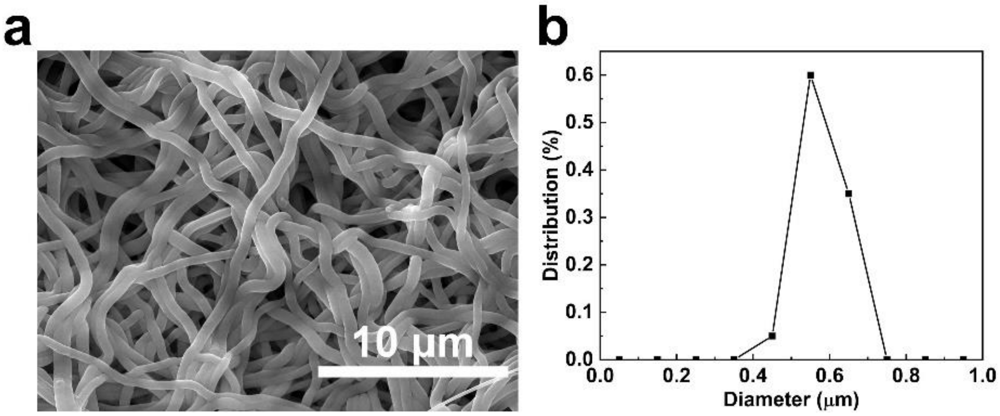


**Fig. S3** **a** SEM images and **b** Diameter distribution of PU-$C_{20}$-3 from transparent to opaque

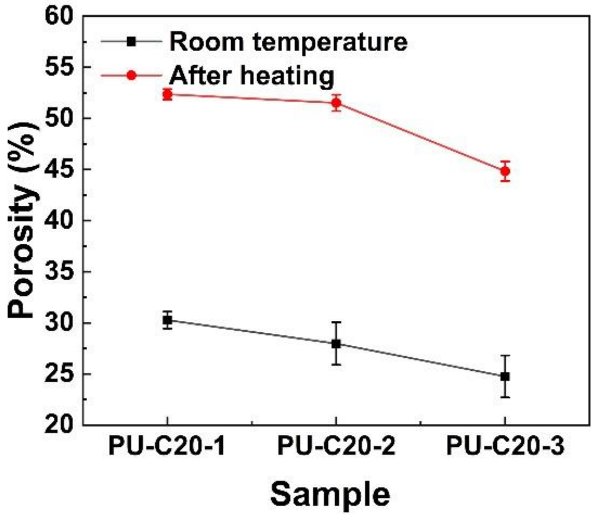


**Fig. S4** Porosity of NFM with different eicosane content

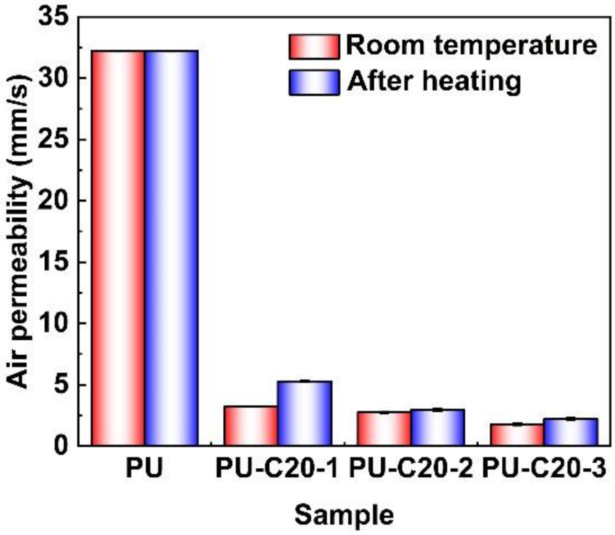


**Fig. S5** Air permeability of different nanofiber membrane materials

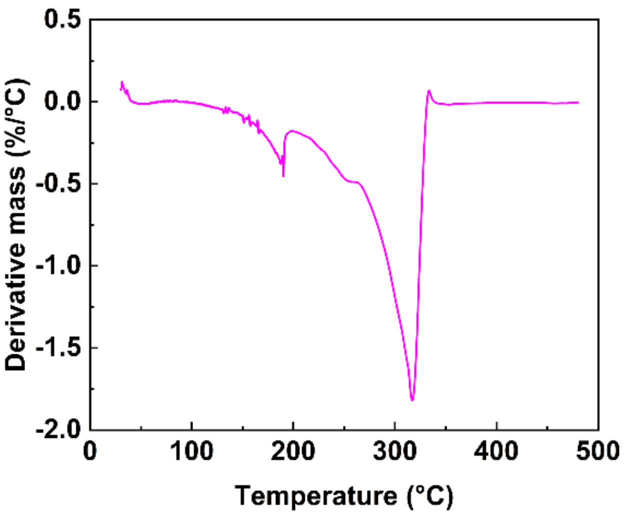


**Fig. S6** DTG curve of eicosane sample

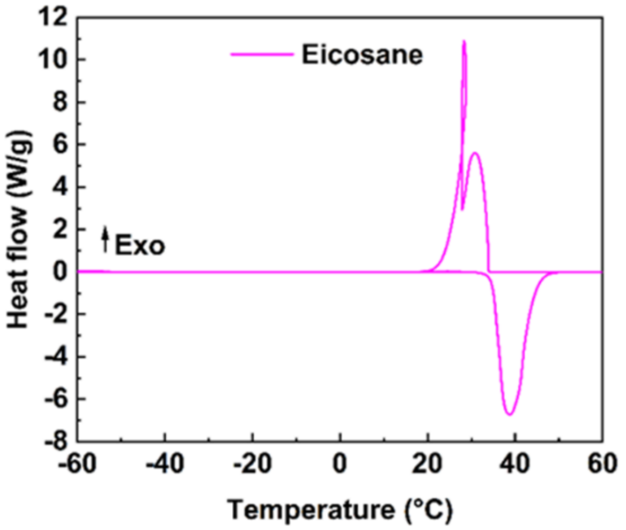


**Fig. S7** DSC curve of eicosane sample

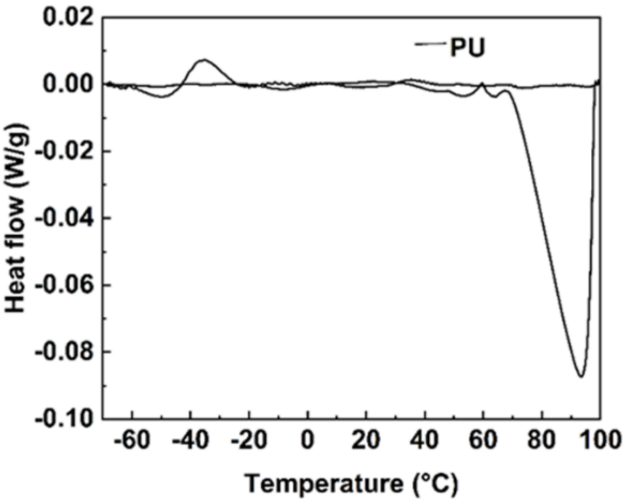


**Fig. S8** DSC curve of the PU membrane sample

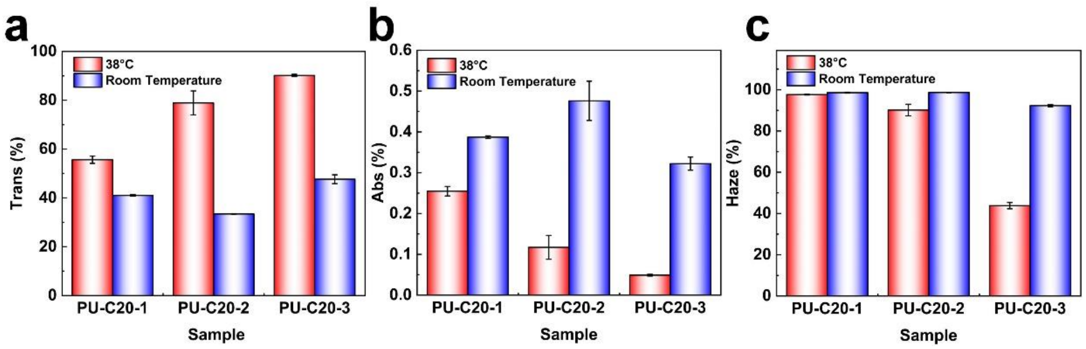


**Fig. S9** Data stability of **a** transmittance, **b** absorbance, and **c** haze of the sample at 38 °C and room temperature

## S4 Supplementary Movies

**Movie S1** Physical demonstration of temperature-responsive transparent nanofiber membranes during temperature changes

**Movie S2** The dynamic microscopic demonstration showcases the temperature-responsive transparent nanofiber membranes at elevated temperatures

## Supplementary References

[S1]H. Chen, A. Baitenov, Y. Li, E. Vasileva, S. Popov et al., Thickness dependence of optical transmittance of transparent wood: Chemical modification effects. ACS Appl. Mater. Interfaces **11**(38), 35451-35457 (2019). https://doi.org/10.1021/acsami.9b11816

[S2]W.W. Dickinson, H.V. Kumar, D.H. Adamson, H.C. Schniepp, High-throughput optical thickness and size characterization of 2D materials. Nanoscale **10**(30), 14441-14447 (2018). https://doi.org/10.1039/c8nr01725e

[S3]W. Zhang, Q. Zhao, S. Puebla, T. Wang, R. Frisenda et al., Optical microscopy–based thickness estimation in thin gase flakes. Mater. Today Adv. **10**, 100143 (2021). https://doi.org/10.1016/j.mtadv.2021.100143

[S4]X. Yang, L. Lan, L. Li, J. Yu, X. Liu et al., Collective photothermal bending of flexible organic crystals modified with MXene-polymer multilayers as optical waveguide arrays. Nat. Commun. **14**(1), 3627 (2023). https://doi.org/10.1038/s41467-023-39162-5

[S5]M. Balasubrahmaniyam, A. Simkhovich, A. Golombek, G. Sandik, G. Ankonina et al., From enhanced diffusion to ultrafast ballistic motion of hybrid light-matter excitations. Nat. Mater. **22**(3), 338-344 (2023). https://doi.org/10.1038/s41563-022-01463-3

[S6]P.J. Burke, Demonstration and application of diffusive and ballistic wave propagation for drone-to-ground and drone-to-drone wireless communications.

Sci. Rep. **10**(1), 14782 (2020). https://doi.org/10.1038/s41598-020-71733-0

[S7]D. Du, X. Jin, R. Deng, J. Kang, H. Cao et al., A boundary migration model for imaging within volumetric scattering media. Nat. Commun. **13**(1), 3234 (2022). https://doi.org/10.1038/s41467-022-30948-7

[S8]Y.R. Lee, D.Y. Kim, Y. Jo, M. Kim, W. Choi, Exploiting volumetric wave correlation for enhanced depth imaging in scattering medium. Nat. Commun. **14**(1), 1878 (2023). https://doi.org/10.1038/s41467-023-37467-z
